# Analysis of the Direct and Indirect Effects of Nanoparticle Exposure on Microglial and Neuronal Cells In Vitro

**DOI:** 10.3390/ijms21197030

**Published:** 2020-09-24

**Authors:** Jasna Lojk, Lea Babič, Petra Sušjan, Vladimir Boštjan Bregar, Mojca Pavlin, Iva Hafner-Bratkovič, Peter Veranič

**Affiliations:** 1 Group for Nano and Biotechnological Applications, Faculty of Electrical Engineering, University of Ljubljana, Trzaska cesta 25, 1000 Ljubljana, Slovenia; babic.lea994@gmail.com (L.B.); vladimir.bregar@kolektor.com (V.B.B.); mojca.pavlin@fe.uni-lj.si (M.P.); 2 Institute of Biophysics, Faculty of Medicine, University of Ljubljana, Vrazov trg 2, 1000 Ljubljana, Slovenia; 3 Department of Synthetic Biology and Immunology, National Institute of Chemistry, Hajdrihova ulica 19, 1000 Ljubljana, Slovenia; petra.susjan@ki.si (P.S.); iva.hafner@ki.si (I.H.-B.); 4 EN-FIST Centre of Excellence, Trg Osvobodilne fronte 13, 1000 Ljubljana, Slovenia; 5 Institute of Cell Biology, Faculty of Medicine, University of Ljubljana, Vrazov trg 2, 1000 Ljubljana, Slovenia; peter.veranic@mf.uni-lj.si

**Keywords:** nanoparticle, neurotoxicity, ROS, cytokine secretion, microglia, co-culture

## Abstract

Environmental or biomedical exposure to nanoparticles (NPs) can results in translocation and accumulation of NPs in the brain, which can lead to health-related problems. NPs have been shown to induce toxicity to neuronal cells through several direct mechanisms, but only a few studies have also explored the indirect effects of NPs, through consequences due to the exposure of neighboring cells to NPs. In this study, we analysed possible direct and indirect effects of NPs (polyacrylic acid (PAA) coated cobalt ferrite NP, TiO_2_ P25 and maghemite NPs) on immortalized mouse microglial cells and differentiated CAD mouse neuronal cells in monoculture (direct toxicity) or in transwell co-culture system (indirect toxicity). We showed that although the low NP concentrations (2–25 µg/mL) did not induce changes in cell viability, cytokine secretion or NF-κB activation of microglial cells, even low NP concentrations of 10 µg/mL can affect the cells and change their secretion of protein stress mediators. These can in turn influence neuronal cells in indirect exposure model. Indirect toxicity of NPs is an important and not adequately assessed mechanism of NP toxicity, since it not only affects cells on the exposure sites, but through secretion of signaling mediators, can also affect cells that do not come in direct contact with NPs.

## 1. Introduction

In recent decades, exposure to nanoparticles (NPs) has significantly increased due to industrial development, increased motor transportation and increased number of engineered NPs used in different consumer products and biomedical applications [1]. Such NPs can enter and accumulate in the body, where they can cause different health-related problems, as indicated by several in vivo animal studies and human epidemiological studies, showing association between ambient/personal exposure to ultrafine particles and adverse health symptoms [2,3,4], including neurotoxicity. Moreover, the incidence of primary brain tumors, developmental and neurodegenerative diseases has increased in the last decades [5]. The exact etiology of these diseases is unknown, but environmental pollutants, including NPs, are frequently mentioned as one of the risk factors [5,6]. Recent studies reported finding combustion derived magnetite NPs in the brains of Alzheimer’s patients [7,8], but no such studies have been conducted for engineered NPs. Unfortunately, research of the potential health risks of NP exposure lags behind the rapid industrial development and commercialization of nanotechnology.

Although the expected accumulation of NPs in the brain is low due to effective protective barriers, even such concentrations can be problematic owing to the specificity of brain tissue. Due to the limited ability of immune and clearance systems in the brain, NPs tend to accumulate and induce cell stress for prolonged time periods, which can cause chronic oxidative stress and inflammation, potentially leading to neurodegenerative changes [6,9]. However, recent studies showed that NPs cannot cause damage only directly, but also indirectly through changes in cell signaling and cell communication between different cell types [10,11,12], or even through intact cellular barriers [13,14].

Microglial cells are the resident innate immune cells, which play an important role in protection of the brain. Consequently, endocytosis of NPs in mixed neural cell cultures mainly occurs in microglial cells [15,16], which are thus the main responders to the toxic insult. However, prolonged and uncontrolled activation of microglial cells may result in direct toxicity to neurons and astrocytes, mainly through secreted reactive oxygen species (ROS), nitric oxide (NO), cytokines and other signaling molecules [17,18,19].

Microglial cells can also be activated by NPs [20,21,22,23] and through their reaction affect neuronal cells, as suggested by some recent studies. For example, Neubert and coworkers showed that presence of primary mouse microglial cells in direct co-culture with mouse primary neuronal cells reduces neurite damage compared to neurite mono-cultures when incubated with different iron oxide NPs, most probably due to NP removal by microglial cells [24]. On the other hand, Hsiao el al showed that indirect exposure to high concentrations of TiO_2_ NPs (30–100 µg/mL) induced more neuronal damage in co-culture model with LPS activated BV-2 microglial cells compared to direct exposure of neuronal cells alone [25]. Similar results were obtained also for silver NPs [26]. Another study showed that conditioned medium from microglial cells stimulated with high concentrations (500 µg/mL) of TiO_2_ or hydroxyapatite NPs, but not SiO_2_ and iron oxide NPs, reduced the viability of PC12 neuronal cells [27]. Another in vitro study on primary cultures of rat brain striatum, which contained several cell types, indicated that microglial cells exposed to TiO_2_ NPs generated ROS, which induced damage and apoptosis in neuronal cells [28]. These studies stress the importance of cell interactions in physiologically more relevant co-culture models on direct and indirect toxicity of NPs, which are not studied enough.

The purpose of this in vitro study was to analyse the possible direct and indirect toxic effect of NPs in mouse neural co-cultures. Mouse microglial and differentiated mouse neuronal CAD cells were incubated with selected NPs that could potentially reach the central nervous system; polyacrylic acid (PAA) coated cobalt ferrite NP, TiO_2_ P25 and uncoated maghemite (MGH) NPs. In contrast to most existing studies, low (2–25 µg/mL), physiologically more relevant concentrations were used to better assess the potential toxicity that such NPs could represent in vivo. We analysed cell viability and confirmed NP uptake in both cell lines and assessed potential activation of microglial cells through analysis of ROS generation and cytokine secretion. The indirect toxicity was analysed in a microglial/neuronal co-culture using the transwell system.

## 2. Results

The aim of this study was to assess the ability of NPs to cause direct or indirect damage on neuronal cells through activation of microglial cells in vitro. Three types of NPs were selected; uncoated MGH NPs as an example of environmentally present exhaust NPs, PAA coated cobalt ferrite NPs as an example of biomedical NPs [29,30,31] and TiO_2_ P25 NPs as an example of industrial NPs present in different coatings and materials [32]. Low concentrations of NPs (2 and 10 µg/mL) were used in order to more closely resemble possible in vivo exposure and accumulation, respectively, and higher (25 µg/mL) was used to assess possible effect that are too subtle at 2–10 µg/mL and for easier comparison with literature.

### 2.1. Nanoparticle Characterization

NPs were characterized in distilled water and cell culture media used for the experiments. The size of NPs (Z-average, hydrodynamic diameter) and polydispersity index (PDI) increased in cell culture media compared to water dispersions (Table 1, Figure S1), most probably due to higher aggregation triggered by the presence of counter ions and formation of protein corona. Protein corona formation also affected the Zeta potential of NPs. Independently of the effective surface charge of NPs in water suspension (37.5 mV for TiO_2_ P25, −50.5 mV for PAA and 39.1 mV for MGH NPs), the Zeta potential in cell culture medium was negative and ranged from −9 to −18 mV, which is closer to the net Zeta potential of serum proteins (−7 mV) (Table 1). While PAA and MGH NPs remained relatively stable despite slight aggregation in cell culture medium, TiO_2_ P25 NPs aggregated and slowly sedimented. The measurements given in Table 1 thus represent mainly the fraction that remained stable during time frame of the measurement.

### 2.2. Direct Effects of NPs on Cell Viability

To distinguish the direct and indirect effect of selected NPs on neuronal and microglial cell lines, we first determined the direct toxicity of NPs on each cell line separately. To adhere to the co-culture exposure protocols, the viability was assessed after 24 h for microglial cells and after 48 h for differentiated CAD cells. 

**Figure 1:**
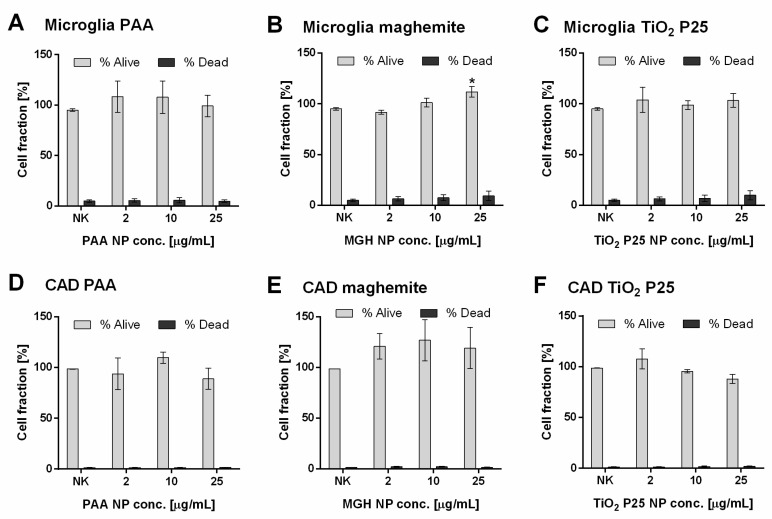
Viability of (**A**–**C**) mouse microglial cells following 24 h incubation and (**D**–**F**) mouse neuronal CAD cells following 48 h incubation with increasing concentration of (**A**,**D**) biomedical polyacrylic acid (PAA) coated cobalt ferrite NPs, (**B**,**E**) uncoated maghemite (MGH) NPs and (**C**,**F**) industrial TiO_2_ P25 NPs. Viability was determined with Hoechst/PI differential staining. Mean and standard error are shown for three independent experiments performed in duplicates. Statistical significance is displayed as: ns (*p* > 0.05); * *p* ≤ 0.05.

There was no decrease in cell viability or significant increase in dead cells (Figure 1) and no changes in cell morphology for neither CAD not microglial cell line (Figures S4–S9). MGH NPs caused a small, concentration dependent increase in cell number in microglial cells (Figure 1B), and a similar, but not significant increase in CAD neuronal cells (Figure 1E). Microglial cells showed a high internalization rate, as indicated by optically dense areas observable inside the cells, especially at higher concentrations of PAA and TiO_2_ P25 NPs (Figures S4 and S6).

### 2.3. NP Internalization (TEM)

**Figure 2:**
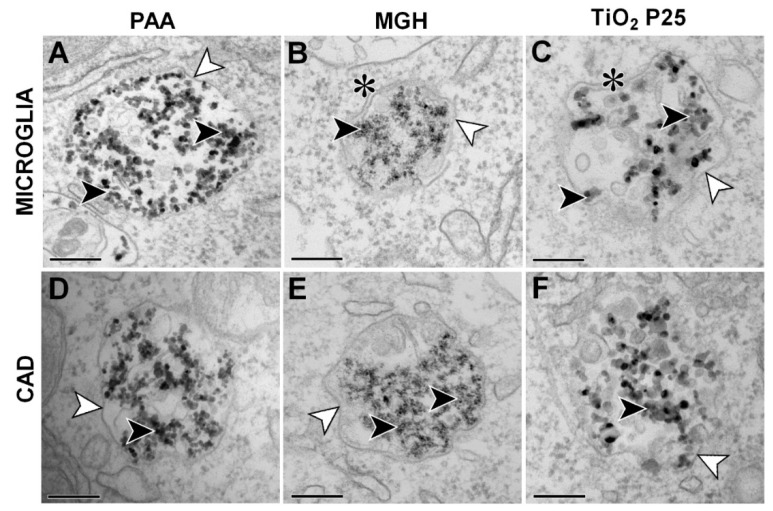
Confirmation of internalization of (**A**,**D**) biomedical polyacrylic acid (PAA) coated cobalt ferrite NPs, (**B**,**E**) uncoated maghemite (MGH) NPs and (**C**,**F**) industrial TiO_2_ P25 NPs in mouse microglial cells (**A**–**C**) following 24 h incubation and differentiated CAD cells (**D**–**F**) following 48 h incubation with 25 µg/mL of NPs. NPs are denoted by full arrowheads and vesicular membranes by empty arrowheads. Double membranes, typical of amphisomes or autophagosomes, are denoted by asterisks. Scale bars correspond to 200 nm.

The internalization of NPs was assessed using TEM following incubation with 25 µg/mL of NPs. All three types of NPs were internalized in both cell lines and were found in membrane bound vesicles undergoing the endolysosomal trafficking route (Figure 2). NPs were observed in endosomes, lysosomes as well as amphysome or possibly autophagosomes, suggesting that the presence of NPs did not significantly interfere with the normal intracellular vesicle trafficking. No NPs were found free in the cytosol or associated with other organelles. Based on the number of observed vesicles on TEM samples, microglial cells showed considerably higher uptake rate compared to CAD neuronal cells (results not shown), which is also consistent with the observations with phase contrast microscopy (Figures S4–S9) and literature [15,16].

### 2.4. NP Induced Changes in Cell Stress and Secretion of Stress and Signaling Molecules

Interactions of microglial cells with NPs can also induce cell stress independent of cell death or decrease in cell viability. Such changes occur on a molecular level and through changes in cell signaling and can result in formation of ROS, NO or secretion of different signaling molecules, such as cytokines. These molecules can then independently of NPs affect neural cells. ROS and cytokine secretion were thus determined for microglial cells incubated with increasing concentrations of selected NPs.

#### 2.4.1. Activation of Cell Stress Response

**Figure 3:**
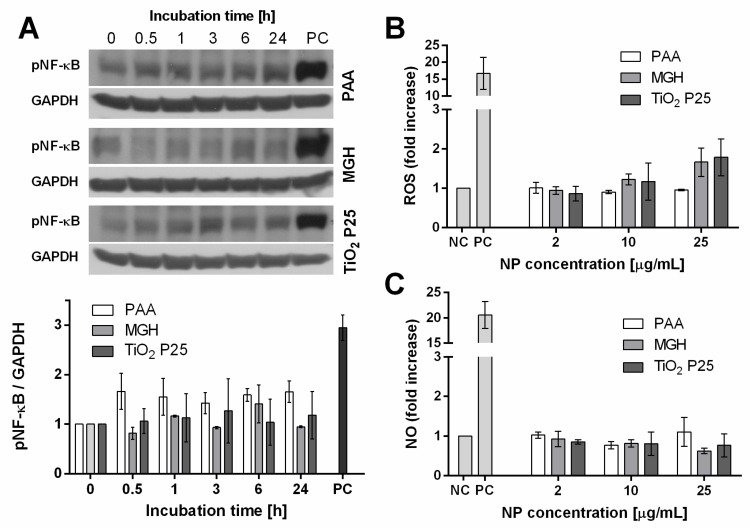
Cell stress response of microglial cells induced by 24 h incubation with increasing concentration of biomedical polyacrylic acid (PAA) coated cobalt ferrite NPs, uncoated maghemite (MGH) NPs and industrial TiO_2_ P25 NPs in mouse microglial cells. (**A**) NF-κB activation was determined at different time points following incubation with 25 µg/mL NPs. 15 min incubation with 0.1 mM H_2_O_2_ was used as the positive control (PC). Mean and standard error are shown for two independent experiments. Representative blots are shown on top. (**B**) ROS were determined with CM-H_2_DCFH-DA assay and normalized for cell number determined by Hoechst 33342 staining. 2mM H_2_O_2_ was used as PC. (**C**) NO measurements were performed with Griess reagent. 5 ng/mL mouse recombinant IFNγ was used as PC. For (**B**,**C**), mean and standard error are shown for three independent experiments performed in triplicates. No statistical differences were detected using One-way ANOVA.

Microglial activation of NF-κB, a central transcription factor in cell stress and immune responses, plays an important role in the release of ROS and pro-inflammatory cytokines that can cause secondary neurotoxicity [33]. Due to the quick dynamics of NF-κB activation, an incubation timeline was performed to observe NF-κB activation dynamics through changes in phosphorylation following 30 min, 1 h, 3 h, 6 h and 24 h incubation with 25 µg/mL of NPs. However, no significant changes were observed on the levels of phosphorylated NF-κB protein for either NP type (Figure 3A).

#### 2.4.2. Generation of ROS and NO

Mouse microglial cells were incubated with increasing concentrations of selected NPs for 24 h and analyzed for increase in generation of ROS and NO. Only the highest tested (25 µg/mL) concentrations of MGH and TiO_2_ P25 NPs induced a (not statistically significant) two-fold increase in ROS in a concentration dependent manner, while the lowest, physiologically achievable concentration (2 µg/mL) had no measurable effects (Figure 3B). PAA NPs had no effect on oxidative stress of microglial cells. On the other hand, none of the chosen NPs increased the secretion of NO following 24 h incubation even at the highest used concentration (Figure 3C).

#### 2.4.3. Cytokine Secretion

**Figure 4:**
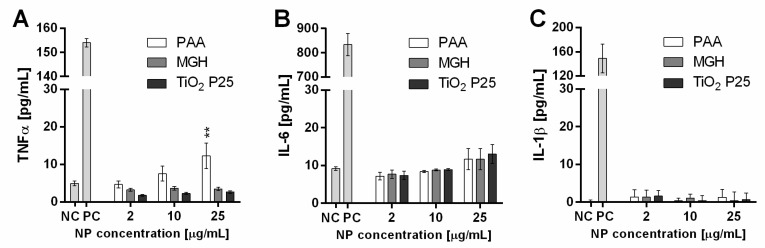
Secretion of (**A**) TNFα, (**B**) IL-6 and (**C**) IL-1β cytokines following incubation with increasing concentration of biomedical polyacrylic acid (PAA) coated cobalt ferrite NPs, industrial TiO_2_ P25 NPs and uncoated maghemite (MGH) NPs in mouse microglial cells as determined with ELISA. For TNFα and IL-6, 100 ng/mL LPS was used as a positive control (PC). For IL-1β, cells were primed with 100 ng/mL LPS for 6 h before exposure and 200 µg/mL crystalline SiO_2_ NPs were used as PC. Mean and standard error are shown for three independent experiments performed in three technical repeats. Statistical significance is displayed as: ns (*p* > 0.05); ** *p* ≤ 0.01.

Microglial cells were analysed for secretion of TNFα, IL-6 and IL-1β, which are the main proinflammatory cytokines secreted from microglial cells and are reported to cause neurotoxicity and chronic inflammatory diseases [19]. There was a minor concentration dependent increase in TNFα secretion following incubation with PAA NPs, while there was no increase in TNFα for MGH and TiO_2_ P25 NPs and no change in secretion of IL-6. There was also no increase in IL-1β secretion, indicating there was no inflammasome activation at 25 µg/mL (Figure 4).

### 2.5. Effects of Secreted Stress Mediators on Viability of CAD Neuronal Cells

To determine the indirect effects of NP exposure on neuronal CAD cells, co-culture experiments with NP exposed microglial cells were performed. For studies of indirect toxicity, we utilized a co-culture model using a transwell system, which enabled simultaneous diffusion of soluble factors thus avoiding the loss of molecules with short half-life, such as ROS or NO, and still analyze each cell line separately. Unfortunately, in this manner we could not asses any effects resulting from direct cell-cell contact, however, due to a significantly shorter generation time of microglial cells compared to differentiated neuronal CAD cells (microglial cells would have overgrown the CAD neuronal cells), direct co-culture would be difficult to perform using the selected cell models.

**Figure 5:**
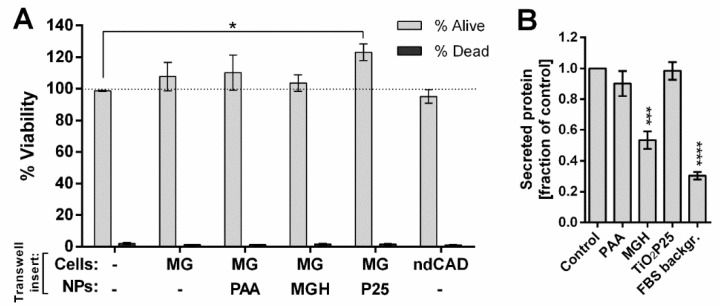
The indirect effects of NPs on mouse neural CAD cells. (**A**) Differentiated CAD cells were incubated with NP exposed microglial cells (MG) for 24 h by using the transwell system. Following incubation with 25 µg/mL NPs, transwell inserts were removed and CAD cells were left to rest for another 24 h in the same conditioned medium before cell viability was assessed microscopically. (**B**) Quantification of proteins secreted by mouse microglial cells incubated with 25 µg/mL NPs for 24 h in FBS free medium. Mean and standard error are shown for three independent experiments. Statistical significance is displayed as follows: ns (*p* > 0.05); * *p* ≤ 0.05; *** *p* ≤ 0.001, **** *p*≤ 0.0001.

The experiments showed that co-culture of differentiated CAD cells with microglial cells induced a slight, non-significant increase in CAD cell number, which was not observed when cells were grown with non-differentiated CAD cells in transwell inserts. Incubation of microglial cells with 25 µg/mL TiO_2_ P25 NPs augmented this effect, while incubation with MGH NPs reduced (not significantly) the observed increase. There was no increase in the fractions of dead cells (Figure 5A). Also, there were no significant changes on cell morphology 48 h after initial exposure (Figure S10).

Since we observed a small effect of microglial response to NP incubation on neuronal cells in co-culture despite the lack of any significant changes in secretion of the measured signaling mediators, we assessed the changes in the total secreted proteins from NP incubated microglial cells. Microglial cells in FBS free medium were incubated with 25 µg/mL NPs for 24 h and the obtained conditioned medium was assessed for the concentration of secreted proteins (Figure 5B) and resolved with SDS-page (Figure S11) to observe any significant changes in quantity of certain protein bands. Incubation with MGH NPs significantly reduced the amount of secreted proteins, while protein secretion from cells incubated with PAA and TiO_2_ P25 NPs remained approximately the same. Only minor changes in specific band intensities can be observed on stained gels (Figure S11), however a more detailed analysis should be performed to determine the significance of such changes.

## 3. Discussion

Although the rate of NP translocation into the brain is relatively low due to effective protective barriers (blood-brain barrier, intranasal mucosa), several studies have shown that environmental and therapeutic NPs can still reach the central nervous system, especially in cases of age-related or pathological damage to the blood brain-barrier. TiO_2_ and iron oxide NPs were detected in mice and rat brain following different routes of administration including inhalation [34,35,36,37,38,39], which is most relevant route for environmental NPs, although other studies failed to detect these NPs in the brain [40,41]. Combustion derived magnetite NPs were also found in the brain of Alzheimer patients at the concentration of 0.2–12 µg/g dry brain tissue [8]. Due to the immunologically restricted status of the brain tissue and a lack of effective mechanism for particle removal, such particles tend to accumulate for prolonged time intervals [42]. This is especially problematic for non-degradable NPs, which can lead to sustained microglial activation and chronic inflammation [33].

Microglia play a double role in toxic insults in the brain: on one hand, acting as phagocytes they remove damaged cells and particulate matter, such as NPs or fibrils, while on the other hand, their activation and secretion of neurotoxic and signaling mediators, such as ROS or cytokines, can damage the neuronal cells, thus contributing to the inflammation and progression of potential neurodegeneration [17,19,33]. This is also reflected in contradicting results obtained in different co-culture studies, where the indirect effects are attributed to the ability of different industrial NPs to activate microglial cells for increased uptake or for secretion of toxic mediators [24,25,26,27].

In this study, we analyzed the ability of low, physiologically achievable concentrations of three types of NPs to activate mouse microglial cells to induce indirect effects on differentiated mouse neuronal cells in vitro. The direct toxicity studies showed that the used NP concentrations (2–25 µg/mL) had no considerable effect on the number of viable cells in either cell line, although there was a slight increase in cell number following incubation with MGH NPs in both cell lines, which could be attributed to the presence of iron ions. A similar tendency was observed also in BV2 microglial cells incubated with iron oxide NPs, depending on their crystal structure [35]. Additionally, iron oxide NPs increased the proliferation and migration of mesenchymal stem cells [43,44], proposing the interference of released iron ions with ROS generation and cell cycle progression as the underlying mechanism. Iron is an important cofactor in several enzymes, including enzymes involved in myelin production and maintenance and mitochondrial oxidative metabolism. As such it plays an important role in cell proliferation, metabolism and differentiation [45,46]. While lower increase in iron concentration might thus have a stimulatory effect on cell proliferation, as observed also in our study, higher concentrations still induce toxicity and possibly neurodegeneration [47,48]. None of the herein tested NPs induced direct membrane damage (Figure 1).

TEM analysis showed internalization of these NPs in both cell lines (Figure 2). Due to the size of NPs and NP aggregates, internalization probably occurred through a non-phagocytic pathway, such as macropinocytosis or clathrin-mediated endocytosis, as observed previously [29,30,49]. Despite significant internalization of NPs in microglial cells, there was no activation of transcription factor NF-κB (Figure 3A) and a low (for PAA NPs) or no increase (for MGH and TiO_2_ P25 NPs) in cytokine secretion (Figure 4). On the other hand, MGH and TiO_2_ P25 NPs induced a small, not-significant concentration dependent increase in ROS, which could be a result of mitochondrial stress or oxidative burst [50]. However, since this was not reflected in NF-κB activation, ROS were most probably generated through the iron-catalyzed Fenton reaction in case of MHG NPs, [51,52] or generated through photocatalytic surface reactivity of TiO_2_ P25 NPs [53].

To assess the effect of NPs on communication between microglial and neuronal cells and possible indirect toxicity of NPs, co-culture experiments were performed. These experiments showed that co-culture of differentiated CAD cells with microglial cells induced a slight, not significant increase in CAD cell number. This increase was not observed when cells were grown with non-differentiated CAD cells in transwell inserts, indicating the increase is most probably caused by signaling/growth mediators constantly secreted by microglial cells and is not a consequence of increased nutrient consumption.

Incubation of microglial cells with 25 µg/mL TiO_2_ P25 NPs slightly augmented this microglia-induced increase in CAD cell number, which was not observed previously for TiO_2_ NPs. Hsiao et al. [25] showed that incubation of murine BV2 microglial cells with 100 μg/mL TiO_2_ NPs (6 nm crystal size, 100% anatase) had no effect on viability of differentiated mouse N2a neuronal cells in a transwell co-culture system. As they also observed no increase in neuronal cell number following co-culture with BV2 microglial cells, the observed increase in neuronal cell number observed in our study and the underlying cell-cell communication might be cell type/cell system specific.

On the other hand, incubation with MGH NPs marginally reduced this microglia-induced increase in CAD cell number. Interestingly, in monoculture experiments, MGH NPs slightly increased the number of CAD cells, while incubation with TiO_2_ P25 NPs reduced their number, clearly indicating that the observed changes on CAD cells in co-culture experiment are a consequence of microglial secreted mediators and not an effect of possible NPs (or iron ions in case of MGH NPs) translocating through the porous membrane to the lower transwell chamber. Since CAD cells were not fully differentiated (the cells still showed a low level of cell proliferation), we assume that the mediators secreted by microglial cells most probably reduced or averted CAD cell differentiation, thus increasing cell proliferation. Moreover, it was demonstrated that low levels of ROS could stimulate proliferation in certain cell types [54], which could also be a possible mechanism behind the increase in neuronal cell number observed following CAD exposure to TiO_2_ P25 stimulated microglial cells, since the later exhibited a 2-fold increase in ROS production at 25 µg/mL TiO_2_ P25 NPs (Figure 3B). Interestingly, the responses of CAD cells to MGH incubated microglial cells correspond to the observed decrease in secretion of soluble protein mediators from microglial cells. The decrease in protein secretion might have occurred through inhibition of the secretory lysosomal pathway, as was observed in a previous study, where Wu and coworkers showed that iron oxide NPs reduced LPS induced secretion of IL-1β (but not TNFα) through impairment of lysosomal degradation and increase in lysosomal permeability [55]. Similarly, Britton and coworkers showed that iron affects the secretion of more than 60 proteins in cultured human adipocytes, including several signaling proteins [56]. Based on the changes in secretion, they propose that iron might have a specific effect on proteins secreted via the classic protein secretion pathway. In this way, MGH NPs and iron ions released from MGH NPs might have reduced the amount of secreted mediators from microglial cell, resulting in the lack of increase in number of CAD cells induced by co-incubation with microglial cells (Figure 5).

To sum up, PAA magnetic NPs, designed for biotechnological and biomedical applications [29,30], expectedly showed the least effects on all analysed cell stress parameters, despite being internalized in the highest amounts in both cell lines. PAA NPs only induces a concentration dependent increase in ROS and TNFα cytokine secretion in microglial cells and no changes were detected on viability of CAD cells in co-culture system. Uncoated MGH NPs, used as an example of environmental NPs, induced a small, concentration dependent increase in viable cells in both cell lines, and a concentration dependent increase in ROS in microglial cells. Despite that, there was a lack of an increase in CAD cell number in co-culture experiments, most probably mediated through MGH induced reduction in secretion of protein mediators from microglial cells. TiO_2_ P25 NPs only induced a concentration dependent increase in ROS, however, an increase in CAD cell number compared to non-incubated CAD cells in co-culture system was observed. Identification of secreted proteins, which would allow us to determine the differentially secreted signaling mediators, as well as changes in gene and protein expression in both cell lines should be performed to better explain and evaluate the observed changes.

Taken together, this study analysed the effect of lower concentration of NPs, which could gain access in central nervous system. We demonstrate that in short time period of 24 h, low concentrations of NPs induce relatively low levels of cell stress responses, and small changes in secretion of protein stress mediators. However, prolonged exposure to NPs and possible accumulation of NPs in cells, as observed in this study even after such short exposure period, could have a cumulative effect exciding the effects observed in this study. Indirect toxicity of NPs is an important and not adequately assessed mechanism of NP toxicity, since it not only affects cells locally (on the exposure site), but through secretion of signaling mediators, can also affect cells that do not come in direct contact with NPs. This is especially important for central nervous tissue due to its restricted repair mechanisms and high susceptibility to inflammation [57]. Moreover, mediators secreted from activated microglial cells can damage neuronal cells, and the damaged tissue further activates microglial cells, leading into a positive activation loop [33]. It is also important to note that NPs can affect the cells through released ions and other impurities, which can, similarly to secreted mediators, translocate the barriers and disrupt the tissue homeostasis away from exposure/accumulation sites. More studies, including long-term studies on relevant co-culture neural models are thus needed to better understand the risks of environmental NP exposure in our daily lives.

## 4. Materials and Methods

### 4.1. Nanoparticles

TiO_2_ P25 NPs were obtained from Sigma-Aldrich (St. Louis, MO, USA), the powder was temperature sterilized and dispersed in ultrapure water. Polyacrylic acid (PAA) coated cobalt ferrite (CoFe_2_O_4_) NPs were prepared as described previously [29]. Uncoated maghemite (MGH) were prepared by precipitation from an aquatic solution of 1M Fe(NO_3_)_3_ and 1 M FeCl_2_ in 90 °C heated 1 M NaOH under vigorous steering for 30 min. The obtained particles were sedimented with a magnet and washed 3 times with dH_2_O, followed by addition of 2M HNO_3_ and mixing for 10 min. The acid was removed by sedimentations, 0.3 M Fe(NO_3_)_3_ was added and heated to 95 °C for 30 min. After cooling the suspension to room temperature and sedimentation, the supernatant was replaced with dH_2_O, mixed and centrifuged for 5 min at 1000 *g*. The supernatant was collected and adjusted to pH 2 with 2 M NaOH to obtain a stable suspension. 

For size characterization and Zeta potential (ξ-potential) measurements of all NPs, we used Malvern Zetasizer Nano ZS (Malvern Industries, Malvern, UK). Zeta potential, hydrodynamic diameter, Z-average size and polydispersity index (PDI) values were obtained. Characterization was performed in distilled water and in DMEM cell culture media with 10% FBS. No presence of endotoxins (<0.5 EU/mL) was found in all NP suspensions (PYROGENT™ Plus Gel Clot LAL Assays, Lonza Group Ltd., Basel, Switzerland).

### 4.2. Cell Culturing, Cell Differentiation and Experiment Design

All experiments were performed on immortalized mouse neural cells. Immortalized microglial cell line was a generous gift from Katherine Fitzgerald and Douglas Golenbock (Department of Infectious Diseases and Immunology, University of Massachusetts Medical School, Worcester, MA, USA), and CAD cell line from Dona M. Chikaraishi (Department of Neurobiology, Duke University Medical Center, Durham, NC, USA). The preparation of immortalized mouse microglial cells from wild type (C57BL/6) mice was described previously [58]. Microglial cells were grown in DMEM cell culture medium (Gibco) supplemented with 10% FBS (Sigma).

Immortalized mouse neuronal cell line CAD, a variant of Cath.a cell line capable of differentiation [59], was grown in OptiMEM supplemented with 10% FBS and adjusted to DMEM supplemented with 10% FBS in order to perform co-culture experiments. CAD cells were differentiated by growing them in differentiation media (DMEM supplemented with 2% FBS and 10 µM retinoic acid; Sigma) for 5 days. All NP incubations were performed in DMEM supplemented with 10% FBS. Both cell lines were grown, differentiated and exposed to NPs in a cell culture incubator in a humidified 5% CO_2_ atmosphere at 37 °C.

### 4.3. Exposure and Co-Culture Protocols

**Figure 6:**
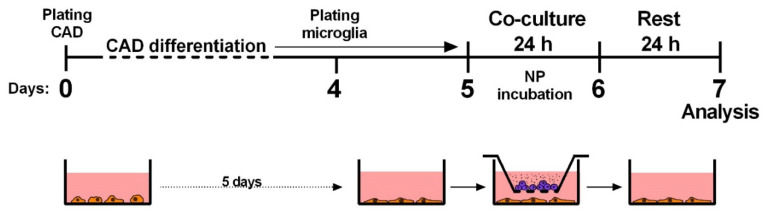
Schematic representation of the procedure for co-culture experiments. CAD cells were differentiated for 5 days, exposed to NP incubated microglial cells in a non-contact co-culture through a transwell system for 24 h and left another 24 h in the same conditioned medium before analysis.

All exposure and non-contact co-culture experiments were performed on microglial cells and differentiated CAD neuronal cells. In co-culture experiments with a transwell system (PET membrane bottom, pore size 0.4 µm, 2 × 10^6^ pores/cm^2^ Sarstedt AG & Co. KG, Nümbrecht, Germany), differentiated CAD cells were exposed to mouse microglial cells incubated with NPs for 24 h. After this, transwell inserts with NP incubated microglial cells were removed and CAD were left another 24 h in the same conditioned medium containing secreted molecules and stress mediators to allow time for manifestation of any effects on cell viability or morphology (Figure 6). To obtain comparable results, each cell line in mono-culture experiments was incubated with the same incubation time as in co-culture experiments: microglial cells were incubated for 24 h and differentiated CAD for 48 h.

### 4.4. Cell Viability Assay

Differential staining viability assay was performed as described previously [29]. Briefly, following NP exposure, cells were stained with 2 μg/mL Hoechst 33342 (Life Technologies, Carlsbad, CA, USA) to obtain the total cell number and with 0.15 mM Propidium iodide (PI; Sigma-Aldrich) for 5 min to stain dead cells. Cells on obtained fluorescent images were counted using CellCounter software [60]. The number of viable cells (*N_S_*) was calculated by subtracting dead (PI positive) from all counted cells (Hoechst positive) for each sample. The percentage of cell viability (% *Viability*) was determined as the ratio between the number of viable cells in each sample (N_S_) and the number of all cells in the non-treated control (N_0_): %Viability = 100 × N_S_/N_0_.

### 4.5. ROS Assay

ROS levels were determined with CM-H_2_DCFH-DA assay (Molecular Probes, Invitrogen, Carlsbad, CA, USA) as described previously [61]. Briefly, cells were incubated with 10 μM CM-H_2_DCFDA in DMEM FluoroBrite (Gibco, Thermo Fisher Scientific, Waltham, MA, USA) at 37 °C for 45 min. We used 0.1 mM H_2_O_2_ as a positive control. CM-H_2_DCFH-DA fluorescence was measured using spectrofluorometer Tecan Infinite 200 (Tecan, Männedorf, Switzerland) and corrected for cell number as obtained with Hoechst 33342 staining. The results are presented as percentage and standard error compared to the negative control sample for three independent experiments in four replicates.

### 4.6. NO Assay

Release of NO following 24 h incubation with NPs was measured with modified Griess reagent (Sigma-Aldricht) following manufacturers instruction. Briefly, supernatants of NP incubated microglial cells were collected and 50 µL of cell supernatant was mixed with 50 µL of 0.04% Griess reagent solution and incubated for 15 min in the dark at room temperature. Mouse IFN-γ recombinant protein (5ng/mL, eBioscience, San Diego, CA, USA) was used as a positive control. For NO quantification purposes standard curve was made from nitrite (Promega, Madison, WI, USA). Absorbance was measured at 570 nm.

### 4.7. ELISA

For detection of mouse cytokines TNFα, IL-6 and IL-1β we used ELISA (Affymetrix eBioscience Inc., Atlanta, GA, USA) following manufacturers’ instructions. 100 ng/mL LPS or 200 µg/mL SiO_2_ NPs (NLRP3 Inflammasome Inducer, Invivogen, San Diego, CA, USA) were used as positive controls. For IL-1β detection, cells were primed with 100 ng/mL LPS for 6 h prior to incubation with NPs.

### 4.8. Transmission Electron Microscopy

Samples for TEM analysis were prepared as described previously [29]. Briefly, microglial cells were incubated with NPs for 24 h before fixation, while CAD cells were first differentiated for 5 days and incubated with NPs for 48 h. Cells were fixed with a mixture of 4% (*w*/*v*) paraformaldehyde and 2% (*v*/*v*) glutaraldehyde in 0.1 M cacodylate buffer, pH 7.4, for 4 h at 4 °C. Post-fixation was carried out in 1% osmium tetroxide in 0.1 M cacodylate buffer for 2 h. Ultra-thin sections examined with a CM100 TEM (Philips Electron Optics, Eindhoven, The Netherlands) for cell ultrastructure, and the analyses of NP uptake and aggregation.

### 4.9. Western Blot

For western blot analysis, cells were washed with ice cold PBS and lysed with 1 × Sample loading buffer [63 mM Tris HCl pH 6.8, 2% SDS, 10% glycerol, 0.002% bromphenol blue, 5% 2-mercaptoethanol]. Protein concentration was measured with Pierce 660 nm Protein Assay Reagent (Thermo Fischer Scientific, Rockford, IL, USA) following the manufacturer’s instructions. Calibrated samples were loaded into 12% polyacrylamide gel, run at constant 30 mA per gel and transferred to PVDF membranes Millipore (Billerica, MA, USA) using the Bio RAD Mini-PROTEAN Tetra Cell (Bio-Rad, Hercules, CA, USA) with constant 150 V for 60 min.

We used primary antibodies against GAP-43 (clone 9-1E12) (#MAB347, diluted 1:1000, Merck, Kenilworth, NJ, USA), Lamin B1 (A-11) (sc-377000, diluted 1:1000, Santa Cruz Biotechnologies, Dallas, TX, USA), p-NF-κB p65 (27.Ser 536) (sc-136548, diluted 1:500, Santa Cruz Biotechnologies) and GAPDH (14C10) (#2118, diluted 1:1000, Cell Signaling Technologies, Danvers, MA, USA) as the loading control. Primary antibodies were followed by incubation with the appropriate HRP-conjugated secondary antibody (Bio-Rad). Bioluminescence was detected with Pierce ECL Western Blotting substrate (Thermo Fischer Scientific). Quantity-One 1-D Analysis Software (Bio-Rad) was used for densitometric analysis.

### 4.10. Analysis of Secreted Proteins

Changes in overall protein secretion were analysed by incubating the cells with 25 μg/mL NPs in FBS free medium for 24 h. FBS background (FBS proteins remaining attached to the plastic following washing steps to remove FBS before incubation in FBS free medium) was obtained by incubating and washing an empty well with the same steps as the seeded wells. Conditioned medium was collected and centrifuge to remove detached cells and NPs. Supernatant was concentrated using Amicon Ultra-0.5 mL Centrifugal Filters 10 kDa NMWL (Merck) and the appropriate amount of 6× Sample Loading Buffer was added. Protein concentration was measured using Pierce 660 nm Protein Assay Reagent following manufacturer’s instructions. Samples were resolved on 4–12% TruePAGE precast gels (Sigma-Aldricht) with constant 150 V. The gel was stained with 0.1% silver nitrate solution and developed with 3% sodium carbonate or with Coomassie blue R-250 following manufacturer’s instructions.

### 4.11. Statistics

Results are presented as mean and standard error. One-way analysis of variance (ANOVA) with Dunnett’s correction for multiple comparisons was performed to test for statistical differences between the control and treated samples. Statistical analyses were carried out with GraphPad Prism v6 (GraphPad Prism Software, La Jolla, CA, USA). Statistical significance is displayed as follows: ns (*p* > 0.05); * *p* ≤ 0.05; ** *p* ≤ 0.01; *** *p* ≤ 0.001, **** *p* ≤ 0.0001.

## Figures and Tables

**Table 1:** Z-average size, hydrodynamic diameter, polydispersity index (PDI) values and Zeta potential of industrial-grade TiO_2_ P25, maghemite (MGH) and polyacrylic acid (PAA) coated cobalt ferrite nanoparticles in distilled water and DMEM cell culture medium supplemented with 10% foetal bovine serum (FBS). Mean and standard deviation are shown for two separate measurements.

	Dispersion Media	Z-Average Size (nm)	Hydrodynamic Diameter (nm) ^a^	PDI	Zeta Potential (mV)
**PAA**	dH_2_O	239.6 ± 41.6	64.5 ± 19.1	0.366 ± 0.009	−50.5 ± 5
	Medium	346.0 ± 49.0	59.5 ± 12.2	0.545 ± 0.057	−17.8 ± 0.6
**MGH**	dH_2_O	128.8 ± 1.1	35.3 ± 3.7	0.196 ± 0.008	39.1 ± 1.9
	Medium	243.9 ± 1.1	67.6 ± 33.6	0.291 ± 0.015	−9.8 ± 0.5
TiO_2_ P25 *	dH_2_O	270.1 ± 6.6	92.3 ± 19.8	0.356 ± 0.002	37.5 ± 0.5
	Medium	406.5 ± 18.6	197.7 ± 10.7	0.324 ± 0.082	−9.3 ± 1.2
**FBS**	Medium	88.8 ± 5.2	8.7 ± 0.7	0 170 ± 0.001	−7 ± 0.2

^a^ Based on the particle number distribution; * TiO_2_ P25 NPs showed a high sedimentation rate. The measurement thus mainly represents the stable fraction of NPs, which did not sediment during the measurement procedure.

## Supplementary Materials

Supplementary materials can be found at https://www.mdpi.com/1422-0067/21/19/7030/s1.

## Abbreviations

NP	nanoparticle
ROS	reactive oxygen species
NO	nitric oxide
LPS	lipopolysaccharide
PAA	polyacrylic acid
MGH	maghemite
PDI	polydispersity index
PI	propidium iodide
TEM	transmission electron microscopy
NF-κB	nuclear factor κB
