# Supplementary material for: Analysis of the Direct and Indirect Effects of Nanoparticle Exposure on Microglial and Neuronal Cells In Vitro
Source: Int J Mol Sci. 2020 Sep 24;21(19):7030. doi: 10.3390/ijms21197030 (PMC7582992; doi:10.3390/ijms21197030)
Supplement: Supplementary file 1 [file ijms-21-07030-s001.pdf]

Analysis of the Direct and Indirect Effects of Nanoparticle Exposure on Microglial and Neuronal Cells In Vitro

Jasna LOJK*†, Lea BABIČ*, Petra SUŠJAN, Vladimir Boštjan BREGAR, Peter VERANIČ, Mojca PAVLIN, Iva HAFNER BRATKOVIČ

† Corresponding author: jasna.lojk@ffa.uni-lj.si

Supplementary information

Nanoparticle characterization

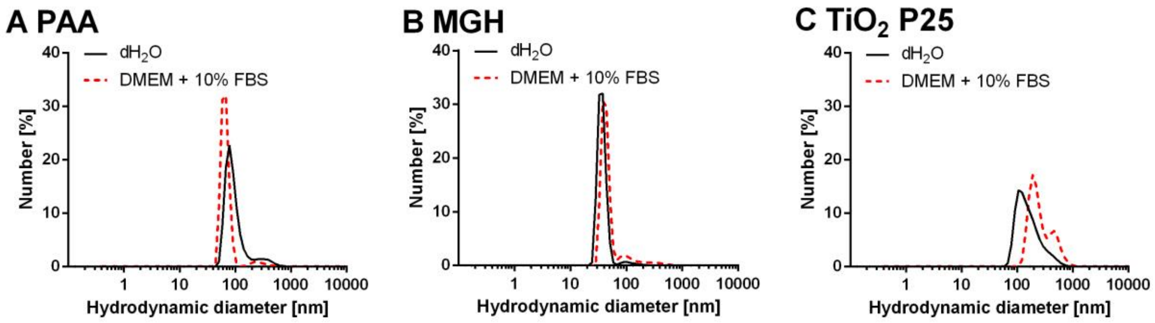

Figure S1: Particle number distribution for (A) polyacrylic acid (PAA) coated magnetic nanoparticles, (B) maghemite (MGH) nanoparticles and (C) industrial TiO_2 P25 nanoparticles analysed in distilled water (dH_2O) and cell culture media supplemented with 10% FBS (medium + 10% FBS). The graphs represent one representative measurement.

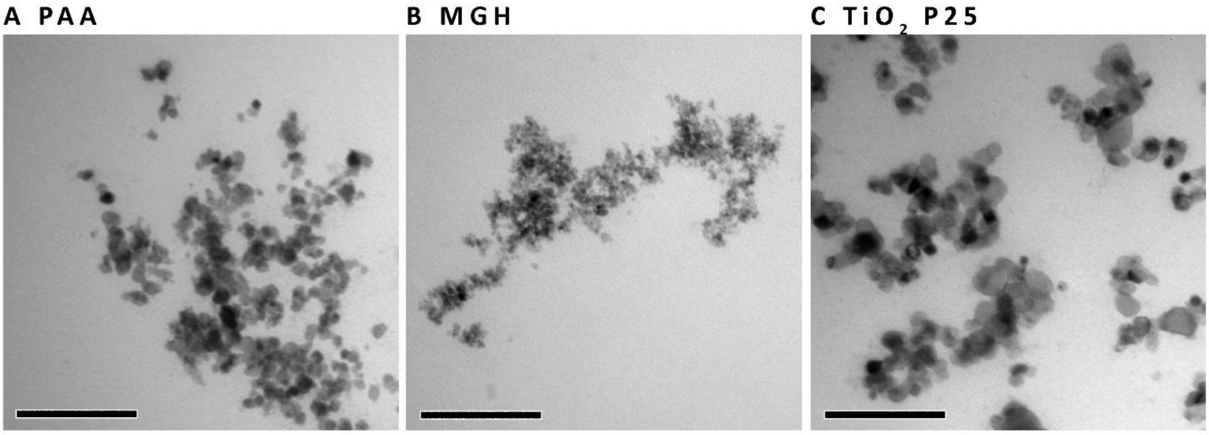

Figure S2: TEM micrographs of (A) polyacrylic acid (PAA) coated magnetic nanoparticles, (B) maghemite (MGH) nanoparticles and (C) industrial TiO_2 P25 nanoparticles in cell culture media with 10% FBS. Scale bars correspond to 200 nm.

CAD cell differentiation

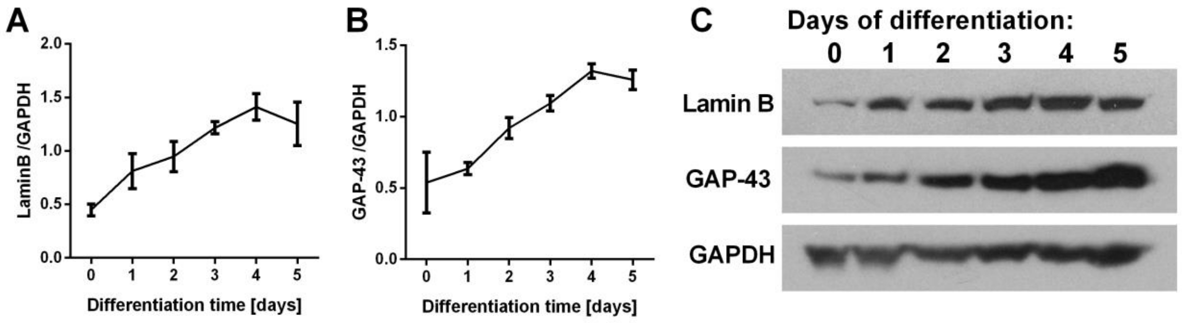

Figure S3: **Differentiation timeline of mouse neuronal CAD cells**. Cells were exposed to differentiation media at day 0 and differentiated up to 5 days. Each day, a sample was lysed to assess changes in neuronal differentiation markers (A) Lamin B and (B) GAP-43. Mean and standard error are shown for three biological repeats. Sample western blot is shown in (C).

Cell viability

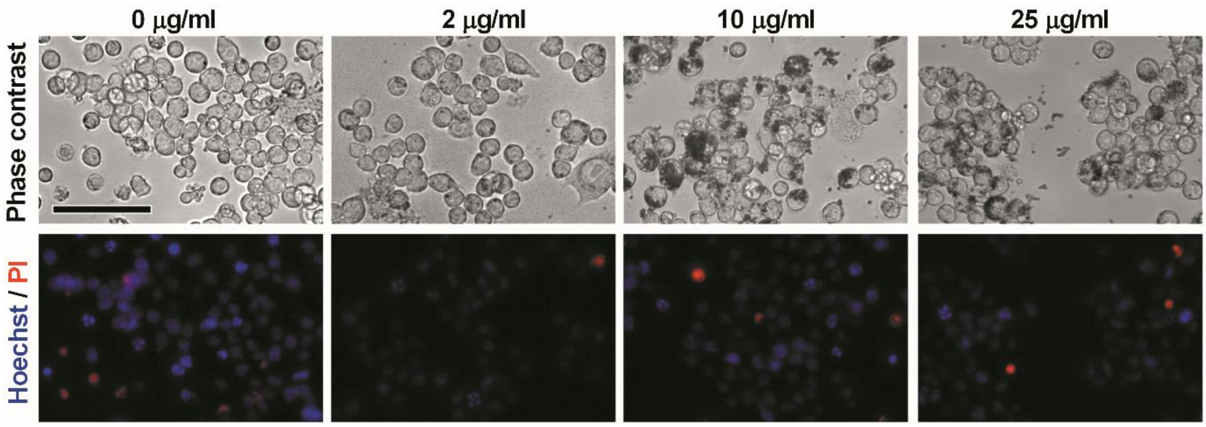

Figure S4: Viability and morphology of mouse microglial cells following 24 h incubation with polyacrylic acid (PAA) coated magnetic NPs. Viable cells are labelled with Hoechst 33342 (blue) and dead cells are labelled with propidium iodide (PI; red). Scale bar corresponds to 50 μm.

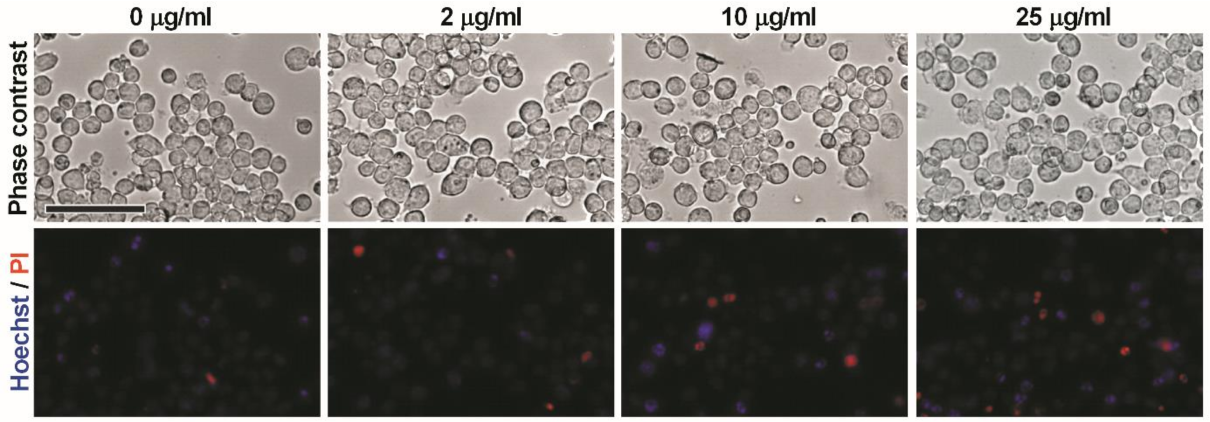

Figure S5: Viability and morphology of mouse microglial cells following 24 h incubation with uncoated maghemite (MGH) NPs. Viable cells are labelled with Hoechst 33342 (blue) and dead cells are labelled with propidium iodide (PI; red). Scale bar corresponds to 50 µm.

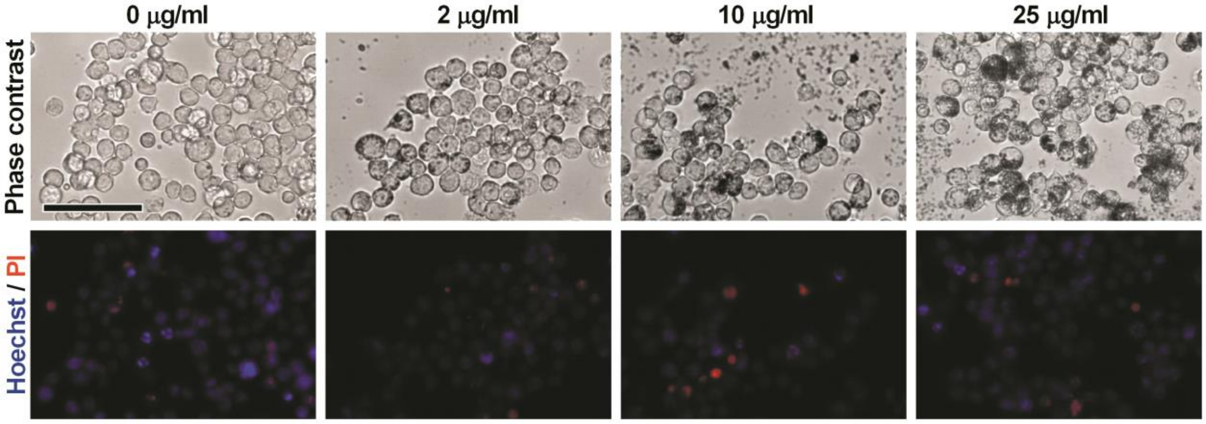

Figure S6: Viability and morphology of mouse microglial cells following 24 h incubation with industrial TiO_2 P25 nanoparticles. Viable cells are labelled with Hoechst 33342 (blue) and dead cells are labelled with propidium iodide (PI; red). Scale bar corresponds to 50 µm.

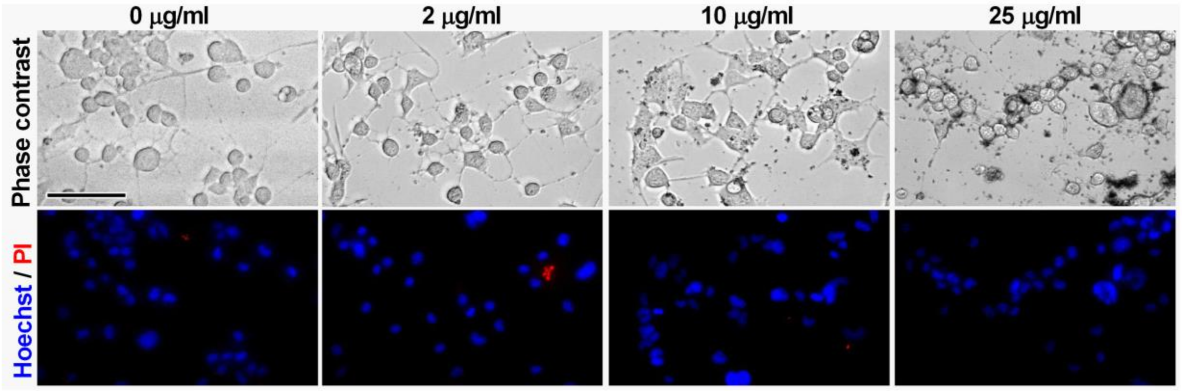

Figure S7: Viability and morphology of CAD neuronal cells following 48 h incubation with polyacrylic acid (PAA) coated magnetic nanoparticles. Viable cells are labelled with Hoechst 33342 (blue) and dead cells are labelled with propidium iodide (PI; red). Scale bar corresponds to 100 µm.

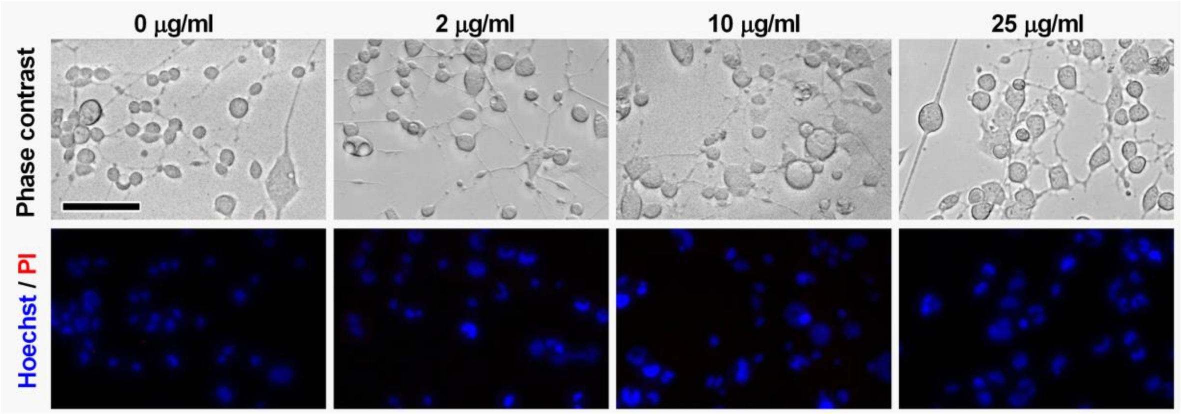

Figure S8: Viability and morphology of CAD neuronal cells following 48 h incubation with uncoated maghemite (MGH) nanoparticles. Viable cells are labelled with Hoechst 33342 (blue) and dead cells are labelled with propidium iodide (PI; red). Scale bar corresponds to 100 µm.

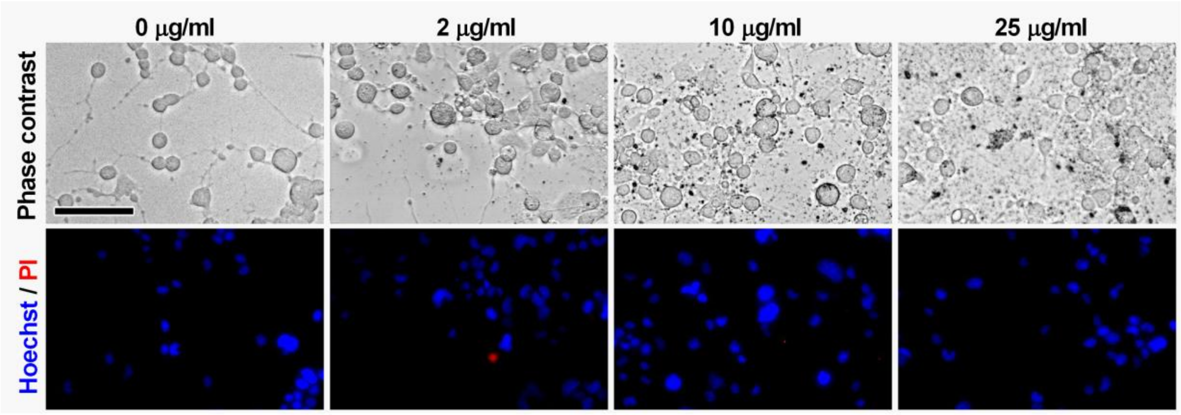

Figure S9: Viability and morphology of CAD neuronal cells following 48 h incubation with industrial TiO_2 P25 nanoparticles. Viable cells are labelled with Hoechst 33342 (blue) and dead cells are labelled with propidium iodide (PI; red). Scale bar corresponds to 100 µm.

Co-culture exposure

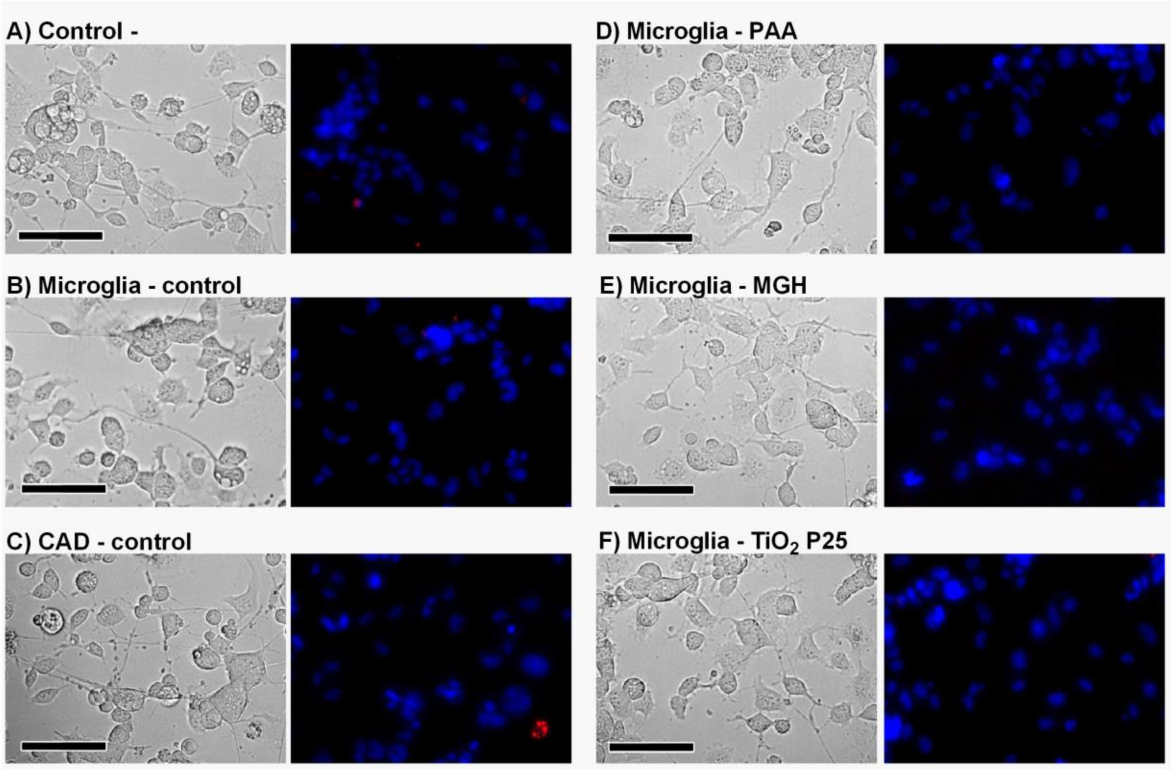

Figure S10: The indirect effect of NPs on viability and morphology of CAD neuronal cells in co-culture with mouse microglial cells. Differentiated CAD cells were either (A) left untreated, (B) grown in co-culture with non-treated microglial or (C) CAD cells as a control, or exposed to microglial cells incubated with 25 µg/mL (D) polyacrylic acid (PAA) coated magnetic NPs (E) uncoated maghemite (MGH) or (F) industrial TiO_2 P25 NPs for 24 h in transwell system. Following 24 h incubation, transwell inserts were removed and CAD cells were left to rest for another 24 h before analysis. Viable cells are labelled with Hoechst 33342 (blue) and dead cells are labelled with propidium iodide (PI; red). Scale bar corresponds to 100 µm.

Analysis of secreted proteins

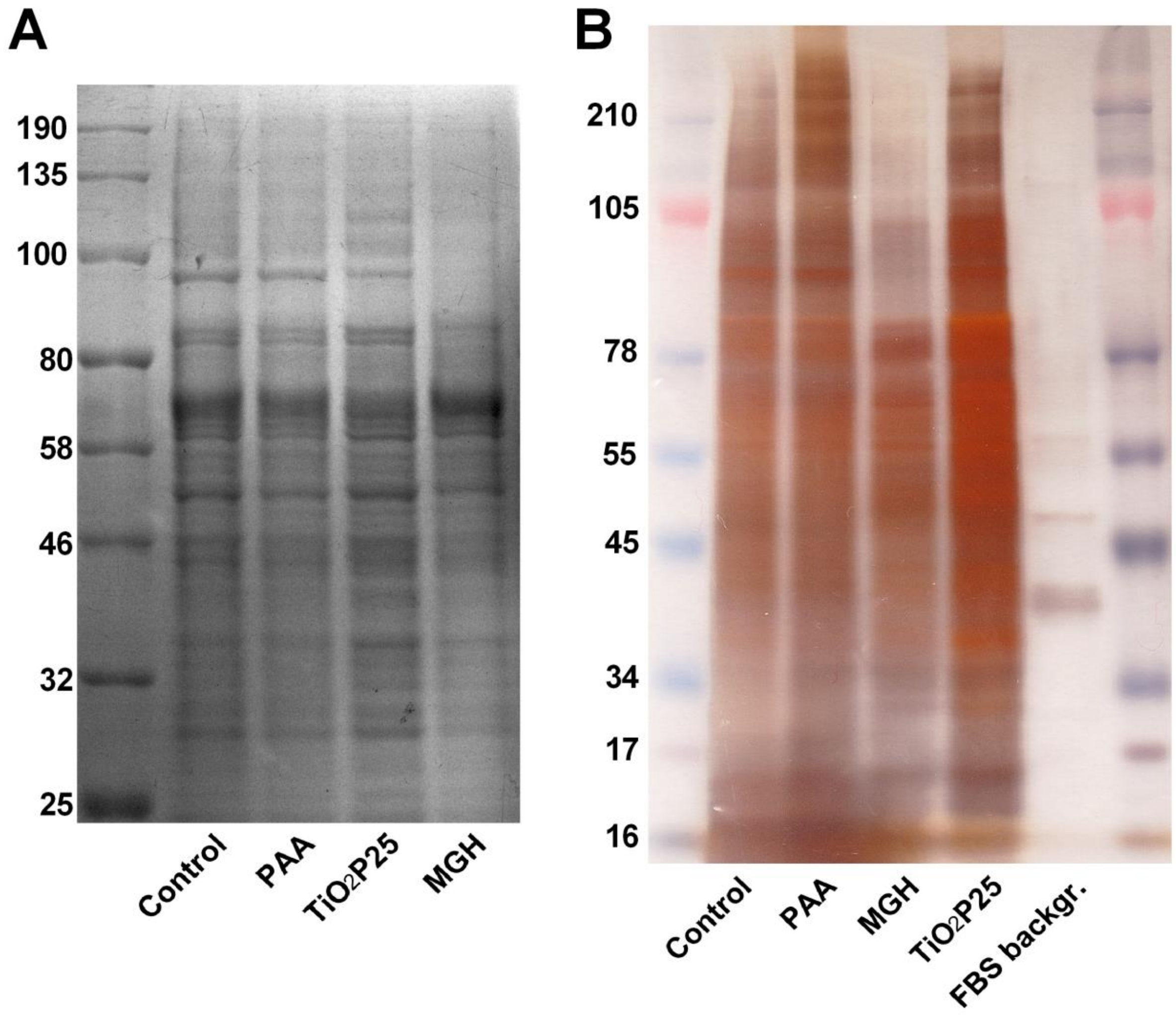

Figure S11: SDS-page analysis of proteins secreted from mouse microglial cells incubated with 25 µg/mL of NPs for 24 h in FBS free medium. Polyacrylic acid (PAA) coated magnetic NPs, uncoated maghemite (MGH) or industrial TiO_2 P25 NPs were used. Untreated microglial cells were used as a control for protein secretion and an empty well with cell culture medium was used to determine FBS background after washing with FBS free medium. Proteins were concentrated and resolved on 4-12% polyacrylamide gels. Gels were stained wither with (A) Coomassie blue stain to obtain low sensitivity staining or with (B) silver nitrate staining to obtain high sensitivity staining.
